# Assessment of the Prediction Power of Forced Ageing Methodology on Lager Beer Aldehyde Evolution during Maritime Transportation

**DOI:** 10.3390/molecules28104201

**Published:** 2023-05-19

**Authors:** Dayana Aguiar, Ana C. Pereira, José C. Marques

**Affiliations:** 1 Faculty of Exact Sciences and Engineering, University of Madeira, Campus da Penteada, 9020-105 Funchal, Portugal; dayana.aguiar@staff.uma.pt (D.A.); jose.carlos.marques@staff.uma.pt (J.C.M.); 2 ISOPlexis, Centre for Sustainable Agriculture and Food Technology, University of Madeira, Campus da Penteada, 9020-105 Funchal, Portugal; 3 Chemical Process Engineering and Forest Products Research Centre, Department of Chemical Engineering, University of Coimbra, Pólo II—Rua Sílvio Lima, 3030-790 Coimbra, Portugal; 4 Institute of Nanostructures, Nanomodelling and Nanofabrication (I3N), University of Aveiro, 3810-193 Aveiro, Portugal

**Keywords:** off-flavours, accelerated ageing, beer exportation, thermal impact, temperature, vibrations

## Abstract

The globalisation of the beer market forces brewers to have methodologies that rapidly evaluate the evolution of beer flavour stability. Commonly used forced ageing methods have limitations since temperature and transportation conditions (temperature, vibrations, long-distance travel, and other factors) impact beer quality. This study assessed the prediction power of a forced ageing methodology on the evolution of aldehydes during maritime transportation across four sample groups (maritime transport, storage simulation, and three ageing periods: 7, 21, and 28 days at 37 °C), which differed in their bottle-opening system (either crown cap or ring pull cap). The results revealed that forced ageing up to 28 days could estimate the evolution of phenylacetaldehyde, 3-methylbutanal, 2-methylpropanal, and hexanal during maritime transport. In contrast, the benzaldehyde content was consistently underestimated, on average, 0.8 times lower. In general, the ageing conditions significantly favoured the formation or liberation from a bound state, up to 2.2 times higher, of *trans*-2-nonenal, acetaldehyde, and 5-hydroximethylfurfural in comparison to the levels registered on exportation simulation beers. Moreover, forced-aged beers with ring pull caps developed quantifiable levels of nonanal and increased phenylacetaldehyde, benzaldehyde, and acetaldehyde content over time. Moreover, thermal stress induced a continuous increase in the extent of beer staling, up to seven times higher, in most samples.

## 1. Introduction

Beer exportation is a continuously growing market, since it is the most consumed alcoholic beverage worldwide. The globalisation of this market represents a challenge for the brewing sector, since the product must maintain as much of its freshness and sensorial features as possible until consumption [1,2]. Beer is a sensitive beverage since its physicochemical properties continuously change almost immediately after production ends, especially when bottled beers face improper storage or exportation conditions: exposition to warm temperatures, vibrations, and long-distance travel [1,3,4,5,6,7]. Beer storage under uncooled temperatures (above 20 °C) critically affects beer flavour stability [8]. The principal changes reported were the loss or decline of beer freshness, bitterness, and pleasant flavours (fruity and floral notes), and, simultaneously, stale flavours such as cardboard and musty appear due to the continuous increase in staling aldehydes, in some cases to levels above their respective thresholds. Additionally, transport conditions are more harmful, since recent findings showed that vibrations reinforce the overall loss of beer flavour stability, acting synergistically with the temperature effect [8]. Few studies reported that bottled beers that underwent transportation showed a significant reduction in their bitterness, fruity flavours, and oxygen content and a significant increase, up to three times higher, in the concentration of staling aldehydes [5,6,8,9].

Staling aldehydes form through Strecker degradation, lipid oxidation, and Maillard reaction are the principal contributors to the undesirable and unreversible changes in beer flavour stability of bottled beers [8]. The appearance of these off-flavours in levels that surpass their flavour threshold on bottled beers can compromise the acceptability of the beer brand by the consumers. The chemical pathways or processes behind the continuous increase in these compounds in bottled beer under storage or transport conditions are yet to be entirely understood [10]. During the beer production process, these carbonyl compounds can end up on the final beer, either in their free state or reversible form bound to a bisulphite or cysteine adduct. Due to their non-volatile character, these bound-state aldehydes are not removed by evaporation during wort boiling or reduced during fermentation. Consequently, they may be present in the final beer. In their bound state, they are undetectable in fresh beer; the same is true for their sensory perception [11,12,13]. Some studies reported that those bound state aldehydes could be released to their free form when bottled beers are submitted to inappropriate temperatures or by the synergetic effect of temperature with vibrations during transportation [13,14].

Thus, the brewing industry needs reliable methodologies to quickly assess the changes in flavour stability, namely on the evolution of the aldehyde content of their brands under storage and exportation conditions. Forced ageing is the methodology commonly used by the brewing sector and scientific community to simulate and accelerate the natural ageing process to predict the evolution of beer flavour stability. This procedure usually submits packaged beer to high temperatures during specific periods, which can lead to significant differences in the chemical reaction rates and promote reactions that do not occur during natural ageing conditions [15,16]. For example, Suarez et al. [17] verified that forced ageing conditions tested (60 °C for 7 days) underestimated the natural evolution of ten aldehydes in naturally aged beers (12 months). They observed that the sum of all aldehydes was up to 3.4 times lower in forced ageing compared to naturally aged beer. Consequently, some limitations on drawing parallelism and conclusions from these approaches were reported, since forced ageing methodologies only considered the effect of one transport and storage variable (i.e., temperature) [8,15,16].

Therefore, this research focused on the prediction power of a forced ageing methodology to assess the aldehyde evolution on bottled lager beers that underwent maritime transport and storage simulation in our previous study [9].

## 2. Results and Discussion

### 2.1. Evaluation of Aldehydes through a Forced Ageing Methodology

The evolution of nine staling aldehydes after maritime transport with storage simulation (45 days of travel at 21–30 °C and 1.7 Hz and 75 days at 19–29 °C after transport simulation) in bottled lager beer evaluated in our previous study [9] were compared with the results of three forced ageing periods (7, 21, and 28 days at 37 °C), to assess the prediction power of this methodology for this particular family of compounds during beer maritime exportation. A total of five beer batches were analysed (B1–B5). Next, the impact of the conditions under study and the main findings found for each compound are discussed according to the aldehyde family (a detailed quantification of the analysed aldehydes in each sample group can be found in Supplementary Material Table S1).

#### 2.1.1. Strecker Aldehydes

According to our previous study, the four Strecker aldehydes identified in bottled lager beers tend to increase, up to three times higher, when beers are exposed to maritime transport conditions [9].

**Figure 1:**
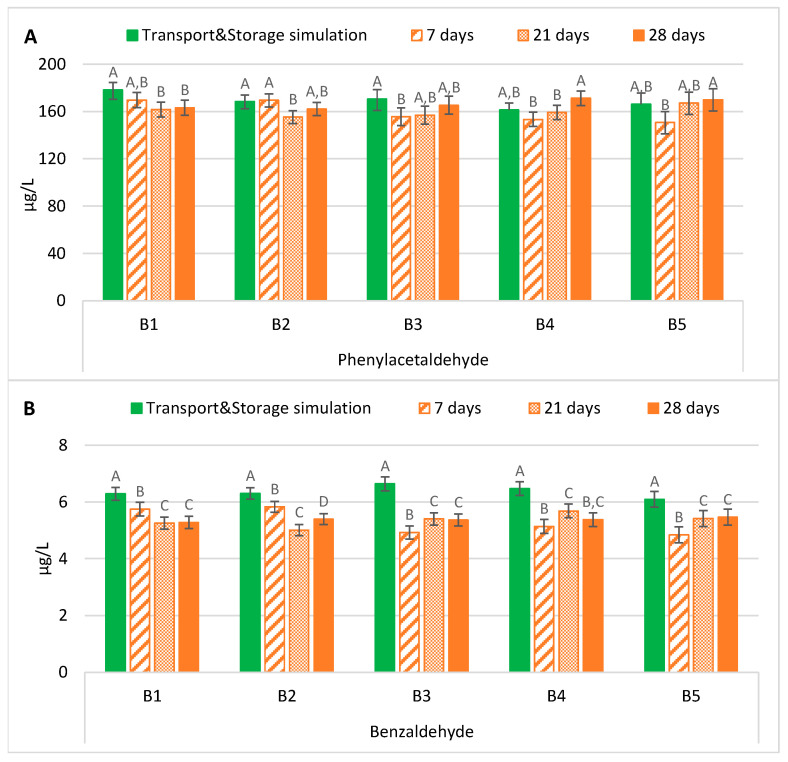
Evolution of Strecker aldehydes under transportation conditions vs. forced ageing: (**A**) Phenylacetaldehyde; (**B**) Benzaldehyde. The Tukey test was carried out per batch. Different letters represent statistically significant differences (*p* < 0.05).

The evolution of phenylacetaldehyde in samples exposed to forced ageing and the evolution of this compound after maritime transport with storage simulation is shown in Figure 1A. The results demonstrated that bottled lager beers forced aged at 37 °C for 7 days or 28 days developed, in general, similar contents of phenylacetaldehyde to those present after maritime transport with storage simulation.

On the contrary, two patterns according to the bottle-opening system were observed on samples aged for 21 days. Bottled beers with crown caps developed significantly lower levels of this aldehyde (*p* < 0.01), on average 0.9 times lower, compared to the levels after transport and storage simulation. On the other hand, samples with ring pull caps had phenylacetaldehyde content that was estimated to make no significant alterations. According to the scientific literature, reported studies pointed out that forced ageing methods have some limitations in mimicking the natural evolution of this Strecker aldehyde. Malfliet et al. [18] reported that lager beers naturally aged for nine months had a phenylacetaldehyde content between 8.2 and 31.2 µg/L, whereas forced-aged beers at 30 °C for 60 days had lower concentrations (5.2–20.2 µg/L). Suarez et al. [17] also concluded that the naturally aged beers developed higher levels of phenylacetaldehyde than forced-aged ones at 60 °C for seven days. After assessing several beers, the authors verified increases from 1.3 to 3.0-fold.

In contrast, it can be observed from Figure 1B that forced ageing at 37 °C for any period tested significantly underestimated (*p* < 0.01), on average 0.8 times lower, the benzaldehyde content developed after maritime transportation with storage simulation.

Two patterns were found when comparing the forced ageing periods tested. We observed a decrease with time in beer samples with the crown cap-opening system, while there was a continuous increase in beers with the ring pull cap-opening system. This effect was possibly due to the diffusion of oxygen through the ring pull cap that, in combination with temperature, catalyses the formation or liberation from a bound state [9]. However, in the latter system, the concentrations were between 14% and 22%, on average, lower than in transport and storage simulation samples. These results were similar to those from the reported study of Malfliet et al. [18]. The authors found that naturally ageing pale lager beers for nine months developed higher levels (1.9–4.4 µg/L) than upon forced ageing at 30 °C for 60 days (1.5–3.3 µg/L). Additionally, Lehnhardt et al. [16] reported that benzaldehyde did not continuously increase during ageing. In another study, naturally aged beers had contents between two and 4.5 times higher than forced-aged beers at 60 °C [17].

**Figure 2:**
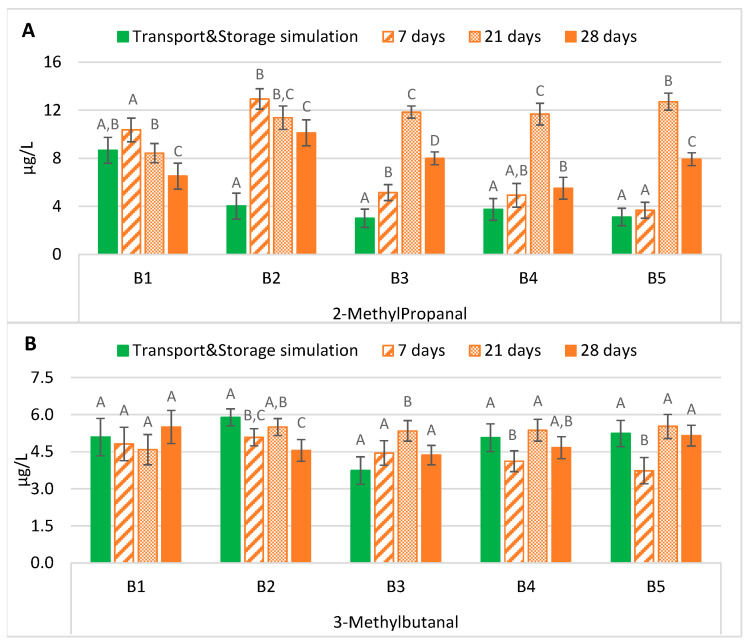
Evolution of Strecker aldehydes under transportation conditions vs. forced ageing: (**A**) 2-methylpropanal; (**B**) 3-methylbutanal. The Tukey test was carried out per batch. Different letters represent statistically significant differences (*p* < 0.05).

The maintenance of lager beers in an oven at 37 °C between 21 and 28 days significantly overestimated (*p* < 0.01), on average three times higher, the actual content of 2-methylpropanal obtained after simulation of the exportation process, mainly in B2 to B5 (Figure 2A). In these batches, the beers submitted to maritime transport and storage simulation had an average concentration of 3.5 µg/L. In contrast, after 21 and 28 days of forced ageing, their average content increased to 11.9 µg/L and 7.9 µg/L, respectively. On the contrary, seven days at 37 °C was the best suitable period to estimate with no significant differences in the content of this aldehyde after simulation of the exportation process. Lehnhardt et al. [16] verified an increase in this aldehyde during ageing at 40 °C for four days, a tendency that generally is verified in the tested conditions (37 °C). The rise in 2-methylpropanal may be strictly related to the valine concentrations in this type of beer. Previous studies (results not published) indicated that this secondary amino acid is present at higher concentrations in bottled beer, which, together with the temperature effect (37 °C), can justify the increase observed in 2-methylpropanal.

As shown in Figure 2B, contrary to the 2-methylpropanal results, forced ageing for 21 or 28 days was the best time to predict the evolution of 3-methylbutanal during the simulated maritime exportation process. Most batches were reproduced without significant differences in the actual content of the beers submitted to maritime transport simulation for almost four months: 5.0 µg/L of 5.1 µg/L (B1), 5.0 µg/L of 5.1 µg/L (B4), and 5.3 µg/L of 5.2 µg/L (B5), respectively (values corresponding to the average of 21 and 28 days of ageing). In the samples from the second batch, only 21 days at 37 °C produced a similar concentration (5.5 µg/L) to the actual value of approximately 5.9 µg/L. On the other hand, in B3, it took 28 days of ageing to simulate the real-world scenario, although the concentration was slightly higher (4.4 µg/L of 3.7 µg/L). Finally, forced ageing for seven days at 37 °C cannot predict the concentration of 3-methylbutanal in beers that underwent to maritime transport simulation.

Based on the description above, to estimate the content of this aldehyde in the studied lager beers after exportation, an ageing period of at least 21 days at 37 °C would be necessary. In most batches, maritime transport and storage simulation beers had a higher content of 3-methylbutanal than after some forced ageing periods, consistent with previous studies [17,18]. Leucine is the precursor of 3-methylbutanal. Previous studies monitoring the amino acid content in this lager beer revealed that leucine was present at residual levels in bottled beer (results not published). Therefore, the *de novo* 3-methylbutanal formation of through this amino acid seems limited.

#### 2.1.2. Evolution of Lipid Oxidation Aldehydes

**Figure 3:**
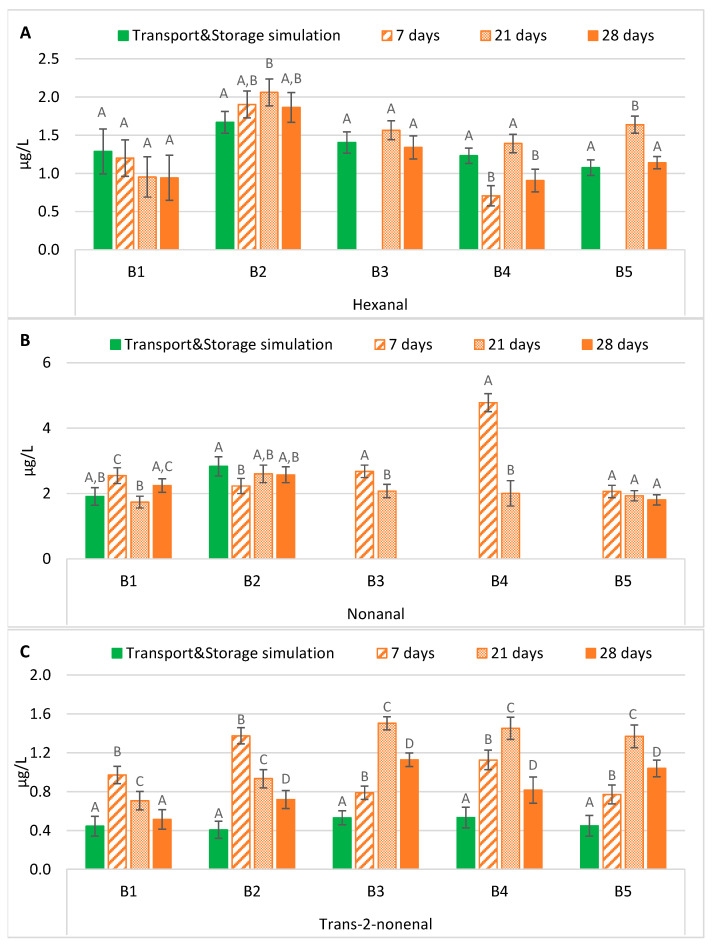
Evolution of lipid oxidation aldehydes under transportation conditions vs. forced ageing: (**A**) Hexanal; (**B**) Nonanal; (**C**) *Trans*-2-nonenal. The Tukey test was carried out per batch. Different letters represent statistically significant differences (*p* < 0.05).

The hexanal content in samples subjected to forced ageing rarely presented significant differences compared to its evolution after maritime transport simulation (Figure 3A). In general, an ageing period of 21 days predicted the evolution of hexanal during the exportation process, regardless of the bottle-opening system. However, forced-aged beers with ring pull caps presented significantly lower levels (*p* < 0.01) after an ageing period of seven days. In contrast, beers with crown caps under the same conditions had a similar trend to beers that underwent transport simulation. From all the tested periods, it can be observed that beer storage at 37 °C for 28 days predicted similar levels of hexanal after maritime transport and storage simulation in most batches. These findings varied from the literature since the forced ageing conditions generally underestimate the normal evolution of hexanal during natural beer ageing. The hexanal content was found to always be lower, on average 2.2 or 3.2 times lower, in forced ageing compared to naturally aged beer [17,18].

The nonanal variation is interesting, since there was a clear division of batches that may be related to the sample-opening system, crown cap (B1 and B2) versus ring pull cap (B3, B4 and B5) (Figure 3B). This compound was quantified in all experimental sets of the first two batches, while in the remaining ones, only in the forced-aged samples. For the first two batches, an ageing time of at least 21 days is necessary to predict a concentration close to the concentrations in the maritime transport and storage simulation samples. On the other hand, in the samples with a ring pull cap-opening system (B3, B4, and B5), the nonanal content was below its respective LOQ in the transport with storage simulation samples (as mentioned in our previous study [9]). The same was observed for an ageing period of 28 days on samples of B3 and B4. Therefore, the results of the last three batches indicate that the conditions of forced ageing studied overestimate the evolution of this lipid oxidation aldehyde under exportation conditions, as previously reported [16], favouring the *de novo* formation or liberation from a bound state of nonanal in these lager beers, with no significant differences being observed between forced ageing regimens only in B5.

According to the simulation performed, the actual content of *trans*-2-nonenal in these lager beers never exceeded 0.6 µg/L after approximately four months of maritime transport and storage simulation, as shown in Figure 3C. However, after forced ageing at 37 °C, its concentration increased significantly (*p* < 0.01) up to values 50 times higher than its flavour threshold (0.03 µg/L) [11], varying between 0.7 and 1.5 µg/L (except for B1 samples aged for 28 days). Therefore, storing beers at 37 °C significantly increased this compound’s concentration after any simulated period, possibly due to its release from an adduct [13]. Consequently, this forced ageing procedure overestimated its actual evolution, on average 2.2 times higher. Lehnhardt et al. [16] also concluded that forced ageing techniques significantly affected the aroma profile and analytical indicators. The same author also showed that *trans*-2-nonenal was mainly found under extreme (heated or acidified) conditions, which resulted in significant increases under forced ageing conditions compared to natural ageing, potentially surpassing its odour threshold and conferring cardboard notes to the beer flavour. In contrast, Suarez et al. [17] reported that forced ageing underestimated the natural ageing in all beers analysed. They observed that *trans*-2-nonenal was always lower, on average 7.9 times lower, in forced ageing when compared with naturally aged beers.

#### 2.1.3. Evolution of Acetaldehyde

**Figure 4:**
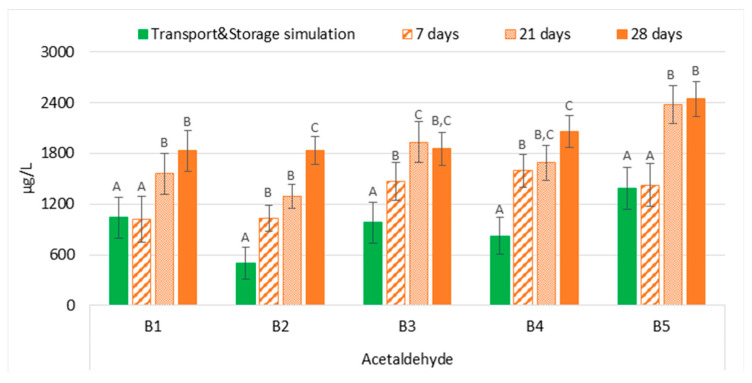
Evolution of acetaldehyde under transportation conditions vs. forced ageing. The Tukey test was carried out per batch. Different letters represent statistically significant differences (*p* < 0.05).

The comparison between the two experimental sets (transport and storage simulation versus forced ageing) demonstrated that the evolution of acetaldehyde in bottled lager beers that underwent transport simulation was generally significantly overestimated (*p* < 0.01) at 37 °C, on average 1.9 times higher, regardless of the ageing period (Figure 4). Therefore, forced ageing at 37 °C cannot predict the acetaldehyde concentration of bottled lager beers submitted to maritime transportation conditions; these results were similar to those reported by Liu et al. [19]. The authors compared natural ageing for up to six months with forced ageing at 60 °C for up to four days. In addition to the continuous increase over time, the authors observed that beers forced aged for four days had a concentration of 4.9 µg/L, which overestimated the acetaldehyde concentration in beers naturally aged for six months (4.3 µg/L). Additionally, the reported continuous increase in acetaldehyde content corresponded to its liberation from a bound state since a simultaneous decrease in diethylacetal levels was detected.

#### 2.1.4. Furanic Aldehydes

**Figure 5:**
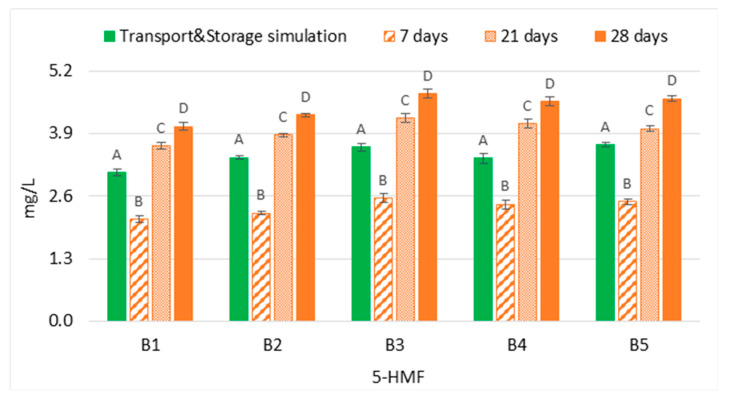
Evolution of 5-hydroxymethylfurfural under transportation conditions vs. forced ageing. The Tukey test was carried out per batch. Different letters represent statistically significant differences (*p* < 0.05).

The content of 5-Hydroximethylfurfural (5-HMF) in both experimental sets (transport and storage simulation versus forced ageing) demonstrated that the evolution of this furanic aldehyde in bottled lager beers that underwent transport simulation cannot be estimated at 37 °C, regardless of the ageing period (Figure 5). The results demonstrated that 7 days of forced ageing significantly underestimated (*p* < 0.01), on average 0.7 times lower, the concentration of 5-HMF when compared to the content reached after transport simulation. On the contrary, a period equivalent to at least 21 days favoured the formation of this compound. Consequently, the content of this aldehyde in bottled beers that underwent transport simulation was significantly overestimated (*p* < 0.01), on average 1.2 times higher. Moreover, a continuous increase across the ageing periods tested was observed since Maillard reactions are favoured at high temperatures [20]. Regarding furfural, this aldehyde was not quantified in any sample of both experimental groups.

### 2.2. TBA

**Figure 6:**
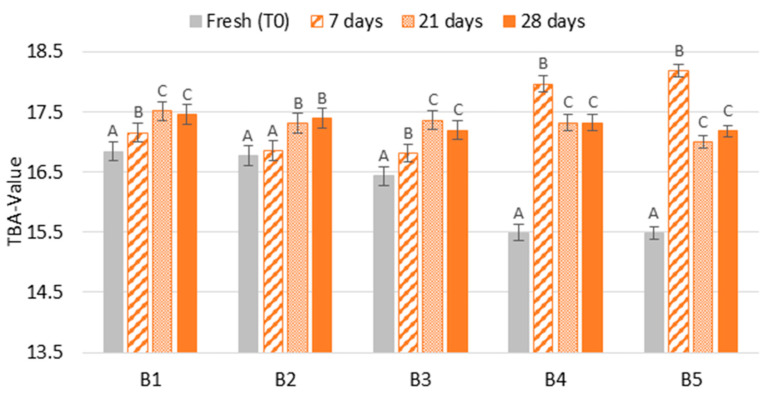
Evolution of beer staling according to TBA-value during forced ageing. The Tukey test was carried out per batch. Different letters represent statistically significant differences (*p* < 0.05).

The degree of thermal stress (TBA-value) of beers submitted to forced ageing compared to fresh beers is shown in Figure 6. The results showed two patterns of evolution. In the first three batches (B1–B3), the TBA-value continuously increased with ageing time, representing an increase between two and seven times compared to forced ageing. For the last two batches (B4–B5—beers with ring pull caps), a significant decrease (*p* < 0.01) was detected after 7 days of forced ageing, meaning that beers forced aged between 21 and 28 days at 37 °C produced up to two times less TBA reactive compounds when compared to beers forced aged for 7 days. Therefore, a continuous beer staling with forced ageing time was observed in the first three batches. Mikyška et al. [21] also observed that TBA-value increased by 89% after three months of storage at 20 °C.

## 3. Materials and Methods

### 3.1. Chemicals and Materials

All chemicals used had a purity grade higher than 95%. Hexanal, benzaldehyde, 2-methylpropanal, nonanal, 2-methylbutanal, 3-methylbutanal, and 4-fluorobenzaldehyde standards were purchased from Sigma-Aldrich (Steinheim, Germany). Acetaldehyde, phenylacetaldehyde, 5-(hydroxymethyl) furfural, 2-furaldehyde, and *trans*-2-nonenal were purchased from Acros Organics (Geel, Belgium). Absolute ethanol, acetonitrile, and methanol (HPLC grade 99.99%) was obtained from Sigma-Aldrich (Steinheim, Germany). Sodium chloride was obtained from Panreac (Barcelona, Spain). Ultra-pure water with a resistivity of >18 MΩ.cm (type 1) was obtained from a Millipore Simplicity^®^ UV apparatus (Milford, MA, USA). The alkane solution (C7–C30) was obtained from Supelco (Sigma Aldrich, St. Louis, MO, USA).

### 3.2. Setup of Transport Simulation and Forced Ageing Experiments

This study aimed to evaluate the prediction power of a forced ageing methodology on the evolution of aldehydes present in lager beer during maritime transportation. A local brewery kindly donated the lager beer samples. This lager beer had an alcohol content of 5.1% [22]. Two experimental setups were performed and compared to accomplish the goal. Maritime transport and storage simulation was described in our previous study [9]. Briefly, 15 commercial lager beers were exposed for 45 days to temperatures between 21 °C and 30 °C with vibrations set at 1.7 Hz to simulate maritime transport. Then, to simulate storage conditions upon arrival at the distributor′s warehouse, the samples were stored in the dark at temperatures between 19 °C and 29 °C for 75 days.

A forced ageing methodology was applied to lager beers to induce beer ageing [23,24]. Only the highest temperature (37 °C) was utilised, since this extreme temperature was outside the temperature range used for the maritime transport simulation. Thus, 45 lager beers (nine samples per batch) were stored in a dark oven at 37 ± 1 °C for 7, 21, and 28 consecutive days.

**Figure 7:**
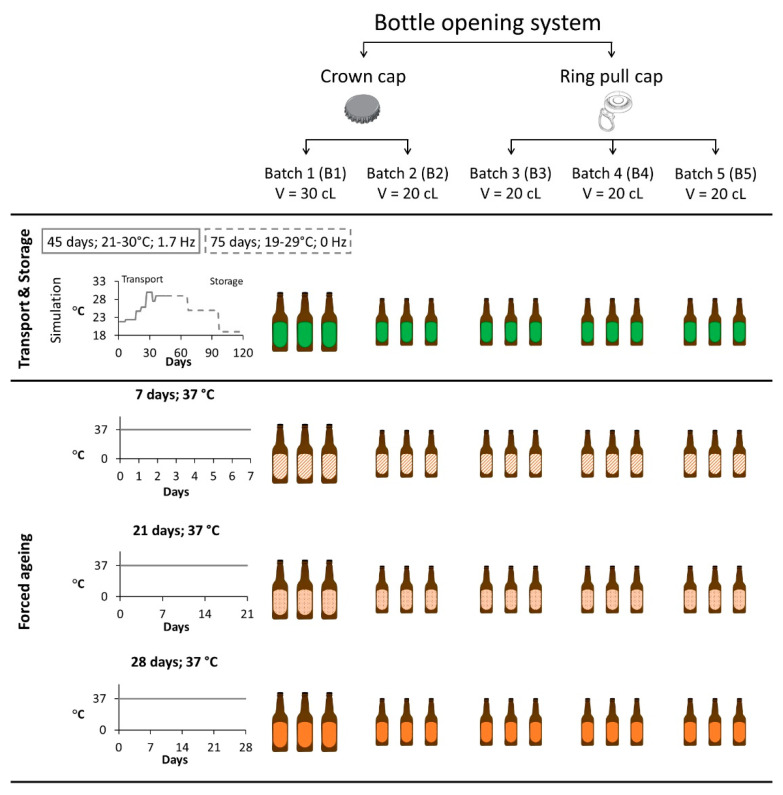
Overview of the transport and storage simulation and forced ageing experimental design.

A total of five different batches were analysed, differing in terms of bottle-opening system (crown caps and ring pull caps) and bottle volume (20 cL and 30 cL). The four sample groups defined to evaluate the methodology prediction power previously described are given in the results and discussion section as follows: (i) transport and storage simulation, (ii) 7 days, (iii) 21 days, and (iv) 28 days. A summary of the number of samples analysed and the conditions that each group experienced are presented in Figure 7.

### 3.3. Quantification of Aldehydes via HS-SPME-GC-MS

Headspace-solid phase microextraction-gas chromatography-mass spectrometry (HS-SPME-GC-MS) was carried out according to the conditions optimized and reported by Vieira et al. [25], with minor modifications, as described in our previous study [9]. Briefly, in each capped glass vial (20 mL), 3.3 g of sodium chloride, 10 mL of beer sample, and 5 µL of the internal standard 4-fluorobenzaldehyde (50 mg/L) were added. Extraction was performed using a Carboxen/Polydimethylsiloxane (CAR/PDMS) fibre coating (85 µm film thickness) for 20 min at 40 °C. The present study performed the extraction in an automatic TriPlus autosampler in SPME mode.

Gas chromatography–mass spectrometry (GC-MS) analyses were carried out using a TRACE GC Ultra gas chromatograph coupled to an ISQ single quadrupole from Thermo Scientific (Hudson, NH, USA). The GC instrument was equipped with a TRB-WAX column (60 m × 0.25 mm) with 0.25 µm film thickness (Teknokroma, Spain). The carrier gas was helium (flow rate 1 mL/min). The injector port was kept at 260 °C, in splitless mode, while the transfer line and the ion source were maintained at 240 °C. The starting temperature was held at 50 °C for 2 min, followed by heating to 100 °C at 3 °C/min, then increasing to 159 °C at 6 °C/min and, finally, to 230 °C at 35 °C/min, where this temperature was maintained for 7 min. The total GC run time was about 40 min. The mass spectrometer was operated in the electron impact (EI) mode at 70 eV. The selective ion monitoring mode was used to analyse the characteristic ions for each analyte. Each sample was analysed in triplicate. Peak detection was performed in Thermo Xcalibur 2.2 software.

### 3.4. Quantification of Furanic Aldehydes by HPLC

The separation and quantification of 5-HMF and furfural compounds were achieved by applying the method proposed by Pereira et al. [26]. An Alliance liquid chromatograph from Waters (Milford, MA, USA) equipped with an auto-injector (Waters 2695) and a photodiode array detector (Waters 2996) system was used. The data acquisition and processing were performed in the Empower Pro software. The chromatographic separation was performed using the following mobile phases: 10 mM phosphate buffer, pH 2.70 with phosphoric acid (A), acetonitrile (B), and methanol (C). The gradient programme varied from 100% aqueous mobile phase to 60% organic phase in 58 min followed by a 12 min re-equilibration. The Atlantis^®^ T3 column (4.6 × 250 mm id; 5 µm; Milford, MA, USA) was thermostated at 30 °C, and the mobile phase was set to a flow rate of 1.0 mL/min.

The compounds were identified by analysing the UV-Vis spectra from 200 to 400 nm, retention times, and spiking samples with pure analytes. Quantification was carried out at 280 nm, according to the external standard calibration curve previously validated. Each sample was analysed in duplicate.

### 3.5. Thiobarbituric Acid Procedure (TBA)

The determination of thermal stress that beer undergoes during forced ageing was performed according to De Schutter et al. [27]. Sample preparation included adding 5 mL of TBA solution (288 mg of thiobarbituric acid in 100 mL acetic acid (90%)) and 10 mL of beer in a test tube. The mixture was kept in a water bath at 70 °C for 70 min. Then, samples were cooled on ice. Finally, the absorbance of the sample was read at 448 nm on a dual-beam Shimadzu UV–Vis 2600 spectrophotometer (Shimadzu, Kyoto, Japan). The analysis was performed in duplicate, using quartz cuvettes with an optical thickness of 10 mm, with untreated beer with TBA solution as blank. Subsequently, the TBA value was calculated using the Equation (1) (D: dilution factor).

(1) $$TBA=10\times \left(D\times A_{448}-A_{blank}\right)$$

### 3.6. Statistical Analysis

The data analysis was conducted using Minitab^®^ 17 (Minitab, LLC, State College, PA, USA), primarily using one-way ANOVA and Tukey test, with a significance level of α = 0.05. This statistical analysis was performed to compare the four sample groups for each batch to determine the prediction power of the temperature-dependant forced ageing method to simulate the evolution of each beer staling aldehydes under study during maritime transportation.

## 4. Conclusions

The application of forced ageing procedures to simulate natural beer ageing should be carefully analysed, especially when the goal is to predict the evolution of a particular family of compounds. In this case, the results demonstrated that forced ageing at 37 °C up to 28 days showed limitations in predicting the evolution of staling aldehydes on beers that underwent maritime transport and storage simulation (45 days of travel at 21–30 °C and 1.7 Hz and 75 days at 19–29 °C after transport simulation). Concerning Strecker aldehydes, generally, the ageing periods tested (up to 28 days at 37 °C) predicted similar levels of phenylacetaldehyde, 3-methylbutanal, and 2-methylpropanal when compared to those verified after maritime transport and storage simulation. The same conclusions were drawn for the lipid oxidation aldehyde hexanal. In contrast, the results showed that the ageing conditions tested significantly favoured the formation pathways or the liberation of these compounds from a bound state, since the levels of *trans*-2-nonenal, acetaldehyde, and 5-HMF were significantly overestimated (up to 2.2 times higher) when compared to their content in beers that underwent maritime exportation simulation. Benzaldehyde was the only Strecker aldehyde that was significantly underestimated, on average 0.8 times lower.

Regarding the bottle-opening system, it was observed that only forced-aged beers with ring pull caps developed nonanal in quantifiable levels. Moreover, it was found that the bottle-opening system appeared to influence the content of some aldehydes in the forced ageing conditions evaluated. For example, the content of phenylacetaldehyde, benzaldehyde, and acetaldehyde continuously increased over time. Lastly, forced ageing conditions assessed promoted a continuous extent of beer staling up to seven times higher for most beer samples.

Despite the adequate prediction for some aldehydes, the forced ageing methods exclusively dependent on temperature presented limitations simulating the evolution of aldehydes in bottled beer under actual transport and storage conditions. Vibrations and more realistic temperatures may contribute to the discrepancies.

## Data Availability

The data presented in this study are available in article or Supplementary Material.

## Supplementary Materials

The following supporting information can be downloaded at: https://www.mdpi.com/article/10.3390/molecules28104201/s1, Table S1: Detailed quantification results of the 12 aldehydes (three samples per batch) in maritime transport simulation and forced ageing (7, 21, and 28 days) samples. Concentration is expressed as mean value ± standard deviation.
