# Supplementary material for: Assessment of the Prediction Power of Forced Ageing Methodology on Lager Beer Aldehyde Evolution during Maritime Transportation
Source: Molecules. 2023 May 19;28(10):4201. doi: 10.3390/molecules28104201 (PMC10222336; doi:10.3390/molecules28104201)
Supplement: Supplementary file 1 [file molecules-28-04201-s001.zip › molecules-2340526-supplementary.pdf]

Supplementary material

Table S1: Detailed quantification results of the 12 aldehydes (three samples per batch) in maritime transport simulation and forced ageing (7, 21, and 28 days) samples. Concentration is expressed as mean value ± standard deviation.

Compound	Batch	Transport & Storage simulation	7 days	21 days	28 days
**Strecker degradation aldehydes**					
Phenylacetaldehyde (μg/L)	1	178.31±8.13^{A}	169.72±9.87^{A,B}	161.69±11.15^{B}	163.26±8.31^{B}
	2	168.46±7.91^{A}	169.47±11.00^{A}	155.28±5.98^{B}	162.11±6.70^{A,B}
	3	170.38±11.20^{A}	155.63±13.18^{B}	156.88±8.03^{A,B}	165.31±11.52^{A,B}
	4	161.28±7.74^{A,B}	153.39±10.96^{B}	159.21±7.53^{B}	171.20±9.29^{A}
	5	166.21±9.37^{A,B}	150.64±19.28^{B}	167.07±16.20^{A,B}	169.85±6.97^{A}
Benzaldehyde (μg/L)	1	6.29±0.31^{A}	5.75±0.30^{B}	5.26±0.21^{C}	5.28±0.41^{C}
	2	6.30±0.33^{A}	5.83±0.37^{B}	5.01±0.22^{C}	5.40±0.22^{D}
	3	6.64±0.52^{A}	4.93±0.20^{B}	5.41±0.32^{C}	5.37±0.19^{C}
	4	6.47±0.32^{A}	5.14±0.19^{B}	5.69±0.42^{C}	5.38±0.46^{B,C}
	5	6.10±0.58^{A}	4.85±0.26^{B}	5.42±0.51^{C}	5.47±0.16^{C}
2-methylpropanal (μg/L)	1	8.67±1.38^{A,B}	10.37±1.16^{A}	8.43±1.24^{B}	6.52±0.68^{C}
	2	4.03±0.58^{A}	12.94±1.22^{B}	11.38±1.36^{B,C}	10.12±1.19^{C}
	3	3.01±0.57^{A}	5.15±0.50^{B}	11.85±0.75^{C}	8.00±0.87^{D}
	4	3.74±0.41^{A}	4.93±0.51^{A,B}	11.68±1.15^{C}	5.51±1.61^{B}
	5	3.21±0.41^{A}	3.68±0.52^{A}	12.72±1.16^{B}	7.92±0.80^{C}
2-methylbutanal (μg/L)	1	nd	nd	nd	nd
	2	nd	nd	nd	nd
	3	nd	nd	nd	nd
	4	nd	nd	nd	nd
	5	nd	nd	nd	nd
3-methylbutanal (μg/L)	1	5.10±1.15^{A}	4.82±0.55^{A}	4.59±0.55^{A}	5.51±0.62^{A}
	2	5.89±0.45^{A}	5.09±5.79^{B,C}	5.50±0.57^{A,B}	4.55±0.34^{C}
	3	3.74±0.59^{A}	4.45±0.35^{A}	5.35±0.68^{B}	4.37±0.46^{A}
	4	5.07±0.52^{A}	4.12±0.52^{B}	5.38±0.63^{A}	4.67±0.74^{A,B}
	5	5.24±0.67^{A}	3.73±0.51^{B}	5.53±0.59^{A}	5.15±0.54^{A}
**Lipid oxidation aldehydes**					
Hexanal (μg/L)	1	1.29±0.17^{A}	1.20±0.42^{A}	0.96±0.19^{A}	0.95±0.14^{A}
	2	1.67±0.18^{A}	1.91±0.19^{A,B}	2.07±0.24^{B}	1.87±0.26^{A,B}
	3	1.41±0.15^{A}	nq	1.57±0.19^{A}	1.34±0.17^{A}
	4	1.24±0.07^{A}	0.71±0.17^{B}	1.40±0.18^{A}	0.91±0.19^{B}
	5	1.08±0.15^{A}	nq	1.64±0.07^{B}	1.14±0.10^{A}
Heptanal (μg/L)	1	nd	nd	nd	nd
	2	nd	nd	nd	nd
	3	nd	nd	nd	nd
	4	nd	nd	nd	nd
	5	nd	nd	nd	nd
Nonanal (μg/L)	1	1.92±0.43^{A,B}	2.55±0.17^{C}	1.74±0.20^{B}	2.25±0.28^{A,C}
	2	2.83±0.32^{A}	2.23±0.27^{B}	2.60±0.33^{A,B}	2.57±0.37^{A,B}
	3	nq	2.68±0.28^{A}	2.08±0.16^{B}	nq
	4	nq	4.78±0.39^{A}	2.01±0.23^{B}	nq
	5	nq	2.06±0.13^{A}	1.93±0.20^{A}	1.81±0.25^{A}
Trans-2-nonenal (μg/L)	1	0.45±0.14^{A}	0.98±0.15^{B}	0.71±0.14^{C}	0.52±0.10^{A}
	2	0.41±0.06^{A}	1.38±0.15^{B}	0.94±0.17^{C}	0.72±0.09^{D}
	3	0.54±0.06^{A}	0.79±0.15^{B}	1.51±0.08^{C}	1.13±0.11^{D}
	4	0.54±0.05^{A}	1.13±0.15^{B}	1.46±0.22^{C}	0.82±0.18^{D}
	5	0.45±0.04^{A}	0.78±0.13^{B}	1.37±0.29^{C}	1.04±0.17^{D}
**Miscellaneous aldehydes**					

Acetaldehyde (µg/L)	1	1038.00±130.21[A]	1021.00±217.96[A]	1559.00±288.50[B]	1831.00±324.46[B]
	2	497.10±172.01[A]	1033.40±149.09[B]	1291.00±203.81[B]	1834.60±178.28[C]
	3	980.30±205.20[A]	1466.00±296.96[B]	1935.00±384.82[C]	1855.60±161.12[B,C]
	4	823.30±138.20[A]	1593.10±149.98[B]	1692.00±410.32[B,C]	2059.30±262.29[C]
	5	1385.90±188.02[A]	1425.00±241.95[A]	2381.00±271.89[B]	2445.60±231.59[B]
**Furanic aldehydes**					
5-hydroxymethylfurfural (mg/L)	1	3.09±0.08	2.12±0.09	3.65±0.09	4.05±0.01
	2	3.40±0.05	2.24±0.03	3.87±0.04	4.28±0.03
	3	3.61±0.09	2.56±0.13	4.21±0.13	4.72±0.04
	4	3.38±0.20	2.43±0.03	4.11±0.07	4.57±0.06
	5	3.67±0.10	2.48±0.02	4.00±0.08	4.62±0.05
Furfural (mg/L)	1	nd	nd	nd	nd
	2	nd	nd	nd	nd
	3	nd	nd	nd	nd
	4	nd	nd	nd	nd
	5	nd	nd	nd	nd

nd - not detected; nq - not quantified
